# Analysis of Drug Release Behavior Utilizing the Swelling Characteristics of Cellulosic Nanofibers

**DOI:** 10.3390/polym11091376

**Published:** 2019-08-21

**Authors:** Sung Won Ko, Ji Yeon Lee, Joshua Lee, Byeong Cheol Son, Se Rim Jang, Ludwig Erik Aguilar, Young Min Oh, Chan Hee Park, Cheol Sang Kim

**Affiliations:** 1 Department of Bionanosystem Engineering, Chonbuk National University, Jeonju 54896, Korea; 2 Department of Mechanical Design Engineering, Chonbuk National University, Jeonju 54896, Korea; 3 Department of Neurosurgery, Research institute of Clinical Medicine, Chonbuk National University Medical School and Hospital, Jeonju 54896, Korea

**Keywords:** polymeric composites, swelling, drug delivery, electrospinning, biomaterials

## Abstract

It is known that the behavior of a drug released from a supporting carrier is influenced by the surrounding environment and the carrier. In this study, we investigated the drug behavior of a swellable electrospun nanofibrous membrane. Nanofibrous mats with different swelling ratios were prepared by mixing cellulose acetate (CA) and polyurethane (PU). CA has excellent biocompatibility and is capable of high water uptake, while PU has excellent mechanical properties. Paclitaxel (PTX) was the drug of choice for observing drug release behavior, which was characterized by UV-spectroscopy. FE-SEM was used to confirm the morphology of the nanofibrous mats and to measure the average fiber diameters. We observed a noticeable increase in the total volume of the nanofibrous membrane when it was immersed in water. Also, the drug release behavior increased proportionally with increasing swelling rate of the composite nanofibrous mat. Biocompatibility testing of nanofiber materials was confirmed by CCK-8 assay and cell morphology was observed. Based on these results, we propose nanofibrous mats as promising candidates in wound dressing and other drug carrier applications.

## 1. Introduction

Over the past years, the importance of nanofibers has increased among researchers, and various studies have been actively carried out using methods such as electrospinning, melt-blown spinning, and spraying. Electrospinning is a conventional and popularly used method of forming polymeric fibers with diameters ranging from 2 nm to several micrometers, which can be controlled by adjusting the voltage, the distance between tip and collector, feed rate, and viscosity [1,2,3]. Nanofibers have been used throughout the industry, and in recent years there has been an increasing trend in use in the field of biomedicine [4,5,6,7]. The properties of the polymer, which is the material of the nanofiber, determine the field to which the nanofiber is applied, and control the function by mixing various polymers and blending them appropriately for the application field. Studies using polymers with properties similar to those of natural materials have also been increasing, particularly polysaccharide-based materials such as cellulose [8,9].

The development of a drug delivery system (DDS) that can control the rate of drug release in specific treatments is the subject of debate in the field of pharmaceutical science and many researchers want to know and control the mechanism of drug delivery [10,11,12,13]. In recent years, there has been an increasing trend toward utilizing polymer properties, structural changes, and stimulus responsiveness to control drug release [14,15,16]. of these is the use of nanofiber mats, which has controlled volume, pore size, and water absorption, and its effect on drug release is examined [17,18]. A three-dimensional scaffold composed of nano-sized hydrophilic polymer can absorb a large amount of water. The wide specific surface area of the nanofibers helps to trap drugs and maintain swelling for the regulation of drug dissolution and diffusion from the scaffold, thereby allowing for long-term sustained drug release to occur [19,20]. In addition, the use of a nanofibrous 3D structure is a reasonable and new structure model with unique properties that can be adjusted according to a specific purpose [21,22,23,24]. From a physicochemical point of view, the ability to modify the structure indicates that these scaffolds can provide different forms of drug delivery. Thus, it can be seen that the higher swelling ratio characteristics of this structure lead to effective control of the drug release.

## 2. Experimental Procedure

Polyurethane (PU, Estane^®^, Skythane^®^ X595A-11, Mw = 110,000, OH, USA) and cellulose acetate (CA, Mw = 30,000) were purchased from Lubrizol Corp. and Sigma-Aldrich (St. Louis, MO, USA) Corp, respectively. A 10 wt % PU solution was prepared by dissolving in a mixed solvent of *N*,*N*-dimethylformamide (DMF, 99.5% Samchun chemical, Pyeongtaek, Korea) and tetrahydrofuran (THF, 99.8% Samchun chemical, Pyeongtaek, Korea) (1:1, wt:wt %). 17 wt % CA solution was prepared by dissolving in a mixture of acetone (99.0% Samchun chemical, Pyeongtaek, Korea) and *N*,*N*-dimethylacetamide (DMAc, Samchun chemical, Pyeongtaek, Korea) (1:2, wt:wt %). The prepared polymer solutions were mixed in different ratios (PU:CA, 10:0, 7:3, 5:5, 3:7 *w*/*w*) for 3 h. We mixed 3% Paclitaxel (PTX, Samyang Genex Corp, Seoul, Korea) per gram of polymer.

The electrospinning system uses a variety of parametric controllable systems and was performed at room temperature (25 °C) and 40–50% humidity. The other electrospinning parameters are listed in Table 1.

We utilized field emission scanning electron microscopy (FE-SEM, SUPRA 40VP, Carl Zeiss, Oberkochen, Germany) to confirm the morphological characteristics of the swellable nanofibrous membrane. The diameters of more than 100 fibers were measured using ImageJ. To evaluate the thermal properties, we performed thermogravimetric analysis (TGA Q20.Q400, Q600.Q800, and TA instrument, Seoul, Korea) that heat rate was set at 10 °C/min and scan from 20 °C to 600 °C. Fourier-transform infrared spectroscopy (FT-IR, Perkine Elmer Co., Waltham, MA, USA) were measured and each spectrum was acquired with a spectral range of 4000–500 cm^−1^.

Identical sizes (20 × 20 mm) of electrospun nanofibrous mats of different PU/CA weight ratios were immersed in 1.0 M phosphate buffer saline (PBS, Sigma-Aldrich, St. Louis, MO, USA) solution for 24 h in a shaking incubator (37 °C, 50 rpm). Percent swelling ratio was determined gravimetrically at various specific time intervals using the following equation [25]:

(1) $$SR\;\left(\%\right)=\;\frac{w_{s}-w_{d}}{w_{d}}\times 100$$

where: *w_s_* = weight of swollen nanofibrous mat at 24 h; and *w_d_* = weight of dried nanofibrous mat.

The thickness of lyophilized samples was measured using a digital vernier caliper while sample morphology and number of thickness through digital vernier caliper was documented via a digital camera.

Standard solutions of PTX with concentrations of 2.5, 5.0, 7.5, and 10.0 mg/mL were prepared using 1.0 M PBS as a solvent. For the release test, 3 wt % of drug loaded PU/CA electrospun nanofibrous mats of different weight ratios was immersed in 1.0 M 10 mL PBS solution. At specific time intervals, an aliquot of the mat-solvent suspension was obtained. The absorbance of both standard solutions and sample aliquots were determined using a UV-Vis spectrophotometer (HP8453, Hewlett Packard, city, Germany) at the characteristic wavelength of PTX of 231 nm. Absorbance reading was done in triplicates for both standard solutions and sample aliquots.

NIH3T3 E1 (fibroblast cell line, Krean Cell Line Bank, Seoul, South Korea) were maintained in Dulbecco’s modified Eagle’s medium (DMEM, Sigma Chemical Co., St. Louis, MO, USA) supplemented with 10% fetal bovine serum (FBS, Sigma Chemical Co., Seoul, South Korea) and 1% penicillin/streptomycin in an incubator at 5% CO_2_ and 37 °C. For cytocompatibility experiments, the cells were cultured in DMEM and seeded into wells with each prepared samples in a 48 well plate at 10,000 cells/cm^2^. Cyrobiocompatibility was evaluated at each time points of 1, 3, and 5 d via the CCK-8 assay according to manufacturer procedures. Briefly, CCK-8 solution as 10% of media was added to each well and incubated with cells for 3 h. Then the optical density of the solution was measured at a wavelength of 450 nm using a microplate reader (Tecan, Männedorf, Switzerland). Triplicate samples were analyzed for each experiment. Afterward to monitor the cell infiltration, cell shape and location of cells on each sample at 1 and 5 d were observed by FE-SEM. The samples were prepared by the process. After culturing the cells in the same way as cytocompatibility experiment, the cells were fixed on samples in 2.5% glutaraldehyde in PBS 4 h at 4 °C and then washed three times with PBS. After that, they were dehydrated with 20, 30, 50, 70, and 100% ethanol solution for 5min each and dried at RT. Each experiment was performed in triplicates (n = 3). Data are presented as mean ± standard deviation (SD). Statistical analysis was conducted based on one-way ANOVA student’s Tukey test (Origin 8.5, USA) and the difference was considered significant at *p* < 0.05(*), *p* < 0.01(**), *p* < 0.001(***).

## 3. Results and Discussion

**Figure 1:**
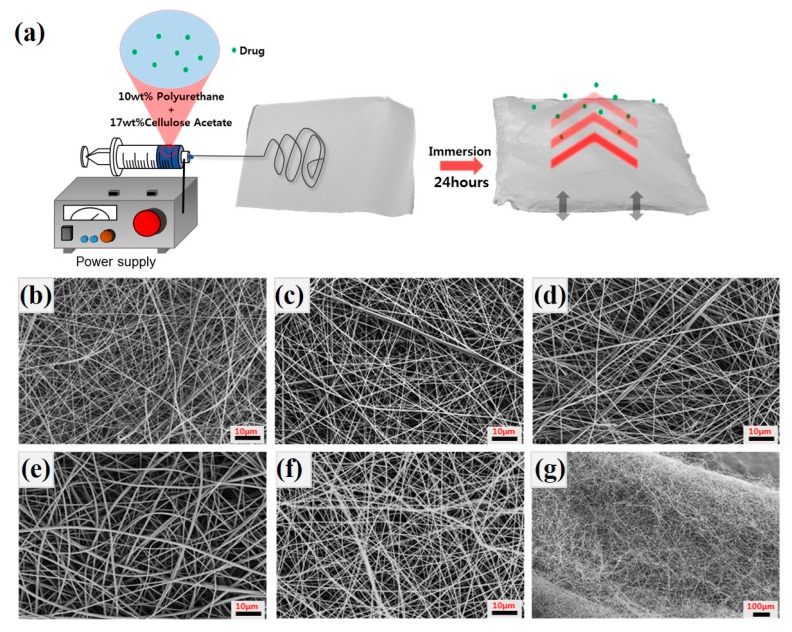
The schematic of overall process (**a**). FE-SEM images of electrospun (**b**) Pure polyurethane (PU), (**c**) PC73, (**d**) PC55, (**e**) PC37, (**f**) PCP37 nanofibrous membrane (scale bar 10 μm), and (**g**) PC37 after swelling test (scale bar 100 μm).

Figure 1a shows the overall schematic of the electrospinning process and behavior of the drug in the swollen nanofiber mat when the electrospun mat is immersed into the aqueous solution. The nanofibrous membrane exhibits a very thin and highly dense morphology. Pure PU and PU/CA composite nanofibers also have similar properties as shown in Figure 1b–f. We describe the diameter of the nanofibers in Table 1, and the diameter of each nanofiber increases as the CA content increases. In the case of PCP373, the diameter of the fiber decreased due to the influence of the drug. After the drug release experiment with the thin PCP373 membrane, lyophilization was performed to observe the morphological changes of the sample as shown in the Figure 1g.

**Figure 2:**
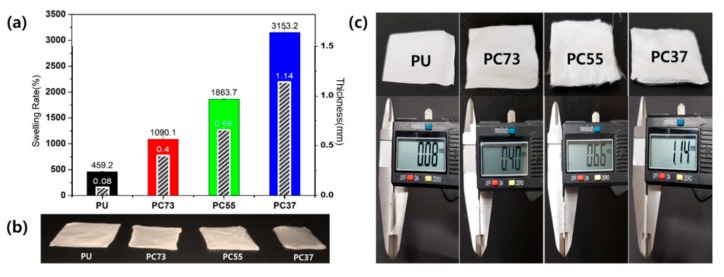
(**a**) Swelling behavior and thickness of PU and composite nanofibrous mats. (**b**) Optical image of the samples after swelling test. (**c**) The lyophilized PU, PC73, PC55, and PC37 membranes and respective thicknesses.

We compared the swelling ratio of nanofibers according to various polymer blending ratios. The apparent difference according to the mixing ratio of the composite is shown in Figure 2a,b, showing the uniform size (20 × 20 mm) samples after a 24-h swelling test. In the case of the swollen PC37 mat, the initial lateral size decreases while the volume increases. The transparency of the composite mat also increased with increasing water content. As shown in Figure 2c, the thickness of lyophilized samples was measured using a Digital Vernier Caliper to compare the vertical volume changes of the samples. The difference between the thickness of PU nanofiber mat and PC37 composites nanofiber was found to be increased by approximately 14-fold.

**Figure 3:**
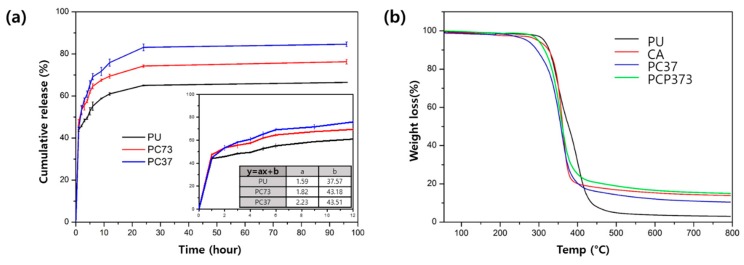
(**a**) Drug release behavior of PU/PC73/PC37 nanofibrous membrane with Paclitaxel (PTX). (**b**) Thermogravimetric analysis (TGA) curves of different composite nanofibers.

For the drug release test, the nanofibrous mats were immersed in PBS solution, and stored in a 37 °C rotation incubator. Drug release experiments were conducted to investigate the drug behavior of these swellable nanofiber membranes. In order to compare the drug release according to the swelling property, three types (PU, PC73, and PC37 with 3 wt % drug) of nanofibers were tested. As shown in Figure 3a, the amount of drug released in the early phase is similar, because the release from the exposed portion of the nanofiber is an important factor in the event of the initial burst release. However, there is a clear difference in the behavior of drug release in the middle-term (after 12 h). There was a difference of about 20% in the amount of drug released after 96 h between the PC and PC37 samples. From this result, we see that drug release behavior depends on the ability of the scaffold to absorb and exchange water. We conducted TGA to evaluate the thermal stability of the composite nanofibers, as shown in Figure 3b. Changes at temperatures below 200 °C are mostly associated with moisture remaining in the fibers. In the case of the PU, if the slope changes at around 370 °C, this is a characteristic curve seen in the PU. The PC37 nanofibrous mat shows a curve associated with the mixing ratio of CA and PU above 400 °C. The TGA curve of the PCP37 nanofiber showed a slight increase in weight loss compared to PC37 due to the drug.

**Figure 4:**
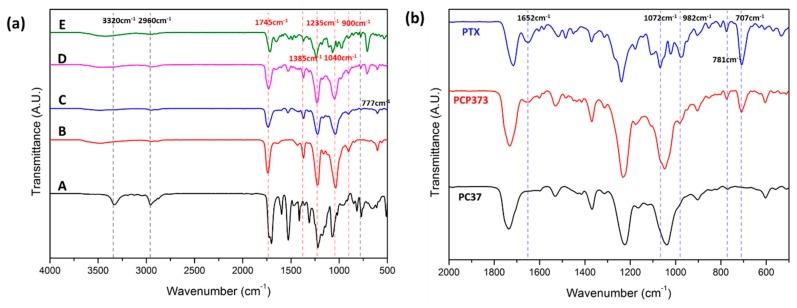
(**a**) FT-IR spectrum of PU(A), PC73(B), PC55(C), PC37(D), CA(E) nanofibrous mats, (**b**) IR spectrum of nanofiber with PTX.

Figure 4 compares specific peaks of the PU/CA composite nanofiber to confirm the synthesis of nanofibers. In Figure 4a, the peaks of Pure PU and CA were compared with the composite nanofiber. For the PU, it has a unique value at 3320, 2960, and 777 cm^−1^ wavenumbers as reported in previous papers [8,26]. CA has characteristic peaks in the spectrum below 2000 cm^−1^, which are 1745, 1385, 1235, 1040, and 900 cm^−1^. CA nanofiber shows a wide peak value at 2900 cm^−1^ and 3200–3700 cm^−1^ [27]. The intrinsic IR peak of PTX has an amide C=O stretch at 1652 cm^−1^. At 1072 cm^−1^, the alkyl C–O stretch of the ester can be confirmed. At 707 cm^−1^, an aromatic C–H bone can be observed (Figure 4b).

**Figure 5:**
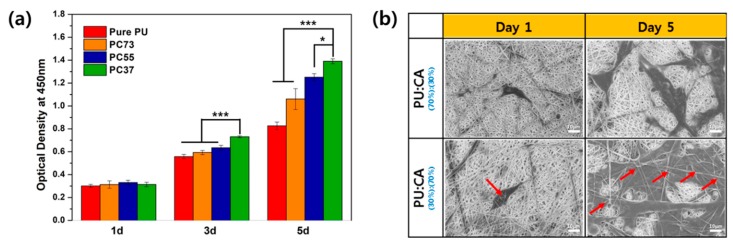
(**a**) CCK-8 assay result of NIH 3T3 fibroblast cell on different membranes and (**b**) SEM image of cell morphology. Data are expressed as mean ± standard deviation (SD), n = 3. (* *p* < 0.05, *** *p* < 0.001 indicates statistically significant difference with PC37).

Biological stability experiments of polymeric materials in contact with human tissues and cells are essential. Figure 5 assessed the biocompatibility of PU and CA composite nanofiber. Experiments were conducted on days 1, 3, and 5. On day 1, there was almost no difference, and from the third day, it was confirmed that the cell proliferation was improved in the sample with high CA content. In the case of the 5th day, it shows a big difference from pure PU.

The topology of the network between NIH 3T3 and nanofibrous membrane is play crucial roles to prove the biological stability during cell propliferation. When the morpological apprearance of the cells on the nanofibrous membrane appears to be long in the lateral direction and attached flat like gum, we assume that the cells are in good condition. Figure 5b shows the morphology of cells in PC37 and PC73 composites nanofibrous membranes. The shape of the cells seen in PC37 of day 5 is similar to that described above, so the cells were in good condition. We also inferred that the swelling ratio is a fact can effect to the entering the membrane layer. The two samples had different CA contents, which means a difference in swelling ratio. When immersed in liquid, membrane pore increased and infiltration of cells into the membrane was observed. The red arrow indicates that the nanofibers are on the cells, and PC73 mat, which has a relatively lower swelling ratio than the nanofibrous mat of PC37, did not show cells entering the layer. Therefore, we were found that the cells were easily enterd into the membrane layer due to the fluffy conformation and swellable property of nanofibrous membrane.

## 4. Conclusions

In conclusion, we succeeded in fabricating drug-loaded swellable composite nanofibrous membranes via electrospinning. It was observed that the mixing ratio of composite nanofibers could control the tendency of swelling rate. On the surface of the swollen nanofiber, morphological changes such as pore expansion were observed. Changes in nanofiber surface morphology were found to affect the drug release kinetics. These results suggest that swellable nanofibers can be applied to the biomedical field.

## Figures and Tables

**Table 1:** Electrospinning parameters and nanofiber diameter.

Sample Name	Concentration (PU:CA)	Drug Content (wt %)	Electrospinning Parameters				Average Fiber Diameter (mm)
			Voltage	Feed Rate	Distance (Tip to Collector)	Needle	
Polyurethane(PU)	10 wt %	-	15 kV	1 mL/h	150 mm	21GA	480 ± 87
Cellulose Acetate(CA)	17 wt %	-					547 ± 156
PC73	(7:3)	-					541 ± 82
PC55	(5:5)	-					593 ± 82
PC37	(3:7)	-					645 ± 81
PCP373	(3:7)	3					603 ± 126
